# An animal paired crossover ePTFE arteriovenous graft model

**DOI:** 10.1186/1750-1164-4-7

**Published:** 2010-11-29

**Authors:** Abdelkarime K Jahrome, Imo Hoefer, Frans L Moll, Graeme J Houston, Peter A Stonebridge, Peter J Blankestijn, Gert J de Borst

**Affiliations:** 1 Department of Vascular Surgery, UMC Utrecht, The Netherlands; 2 Department of Experimental Cardiology, UMC Utrecht, The Netherlands; 3 Tayside Flow Technologies, Dundee, UK; 4 Department of Nephrology, UMC Utrecht, The Netherlands; 5 Department of Vascular Surgery, The Netherlands

## Abstract

**Purpose:**

Previously, we developed a porcine model for Arterio Venous Graft (AVG) failure to allow assessment of new access strategies. This model was limited concerning graft length. In the present technical report, we describe a modification of our model allowing the assessment of long AVGs.

**Technique:**

In 4 pigs, AVGs of 15 cm length were created bilaterally in a cross-over fashion between the carotid artery and the contralateral jugular vein. Two days (2 pigs) and two weeks (2 pigs) after AV shunting, graft patency was evaluated by angiography, showing all four grafts to be patent, with no sign of angiographic or macroscopic narrowing at the anastomoses sites.

**Conclusions:**

In this modified pig AVG failure model, implantation of a bilateral cross-over long AVG is a feasible approach. The present model offers a suitable tool to study local interventions or compare various long graft designs aimed at improvement of AVG patency.

## Introduction

**Figure 1:**
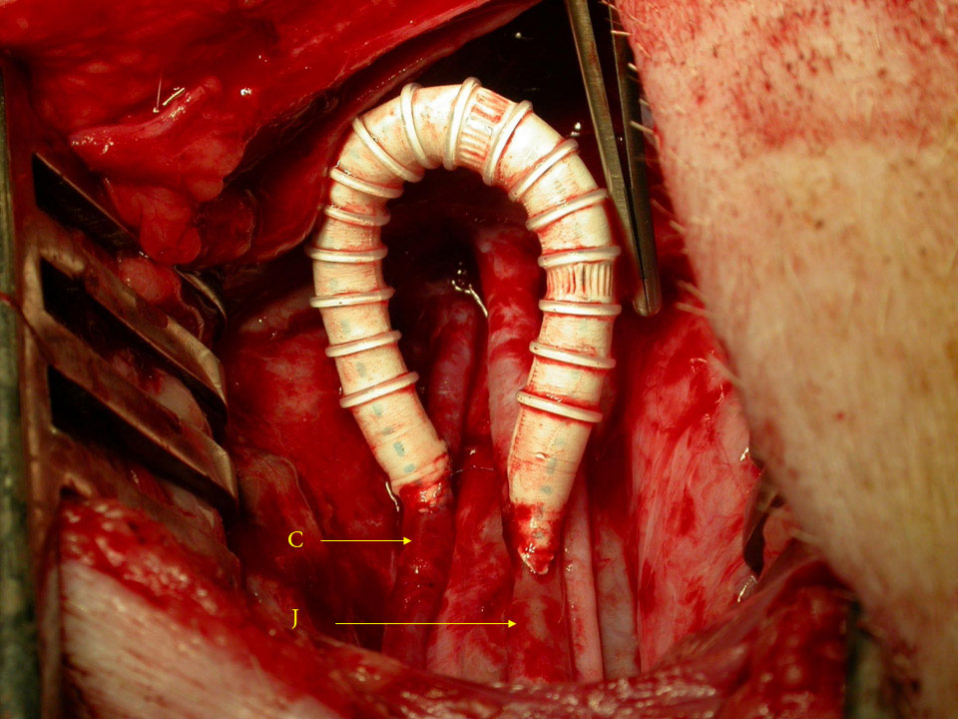
**Carotid - jugular fistula using a short length (7 cm) ePTFE AVG between the common carotid artery and *ipsilateral*internal jugular vein in the original Utrecht Porcine model**. J = jugular vein; C = carotid artery.

Intimal Hyperplasia (IH) in the venous outflow traject constitutes the number one cause of ArterioVenousGraft (AVG) malfunction and failure. Previously, our group described a porcine model for rapid AVG failure due to IH [1].This model consisted of a bilateral short 7 cm length loop between the ipsilateral internal jugular vein and common carotid artery using reinforced, thin-walled, 5 mm inner diameter extended Polytetra fluoro ethylene (ePTFE) grafts (Figure 1). Despite a suitable tool to study local interventions for short length AVG, a serious limitation of this model was the maximum possible graft length of 7 cm due to ipsilateral positioning in the dissected animal neck. Furthermore, AVGs with various design modification such as tapering or the addition of external reinforcements could not be tested.

In the present report, we describe our experience with a modification on the previous model, addressing this limitation, enabling us to implant and study long length AVG.

## Technique

### Animals

A total of 4 female Landrace pigs, weighing 56 ± 2.6 kg, received bilateral crossover AVG (Thin-walled, ePTFE grafts without circular reinforcement measuring 5 mm inner diameter and 150 mm in length (W. L. Gore, Flagstaff, AZ, USA) versus ePTFE spiral graft (Tayside Flow Technologies Limited, Dundee, UK) between the carotid artery and the contralateral internal jugular vein. The rationale for using two grafts types was based in the search by Tayside for a suitable animal to test a new spiral flow bypass. Due to the required length of this graft, we decied to modify our exisiting animal model, using our former basic ePTFE graft as a control. Within this small pilot and feasibility study no further data on the different behaviour of the two graft types were collected other than data as presented in the present technical description. The study protocol was approved by the Institutional Review Board for animal experimentation of the University Medical Center Utrecht and conforms to the *Guidelines for the Care and Use of Laboratory Animals *[2].

### Anesthesia

Before the operation and termination, the animals were fasted overnight and premedicated with intramuscular ketamine 10 mg/kg, midazolam 0.4 mg/kg, atropine 0.5 mg, followed by intravenous thiopental sodium 4 mg/kg. After intubation, animals were mechanically ventilated and anesthesia was maintained intravenously with 0.5 mg/kg/h midazolam, 2.5 μg/kg/h sufentanil and 0.1 mg/kg/h pancuronium. The animals were monitored by electrocardiogram and capnography.

### Antiplatelet therapy

Starting 6 days preoperatively, the pigs received acetylsalicylic acid 80 mg/dd orally. Clopidogrel (Sanofi-Synthelabo, Paris, France) 225 mg was added 1 day preoperatively and continued at a dose of 75 mg/day orally until termination. Heparin 100 IU/kg was provided intravenously before arterial or venous vessel manipulation.

### Operative procedure

**Figure 2:**
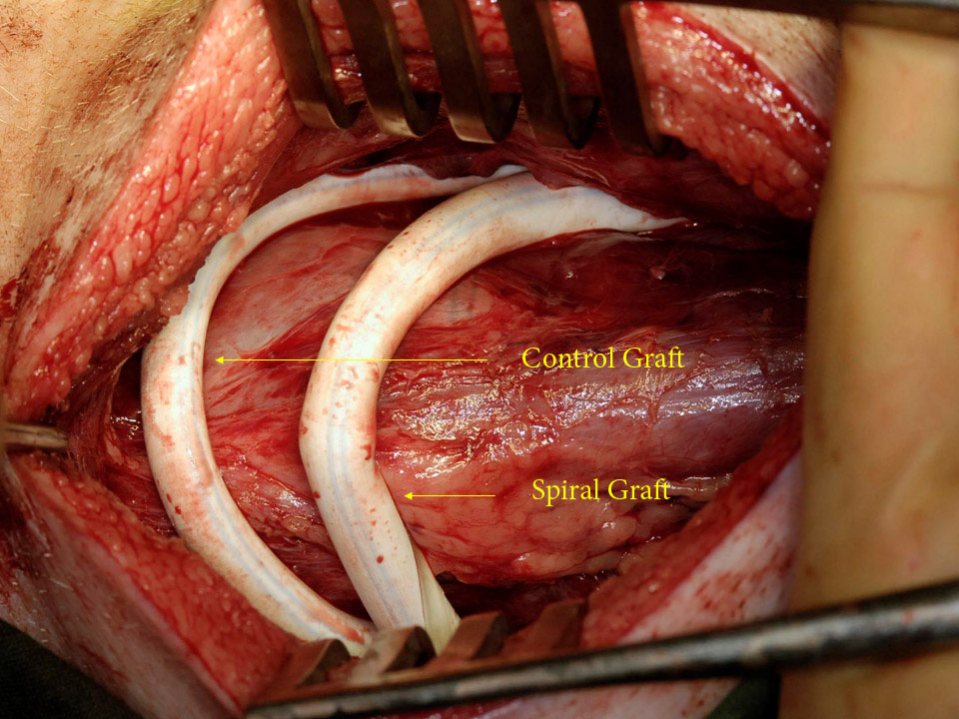
Crossover carotid jugular fistula showing a paired bilateral long length ePTFE AVG from the common carotid artery connected to the *contralateral*internal jugular vein.

**Figure 3:**
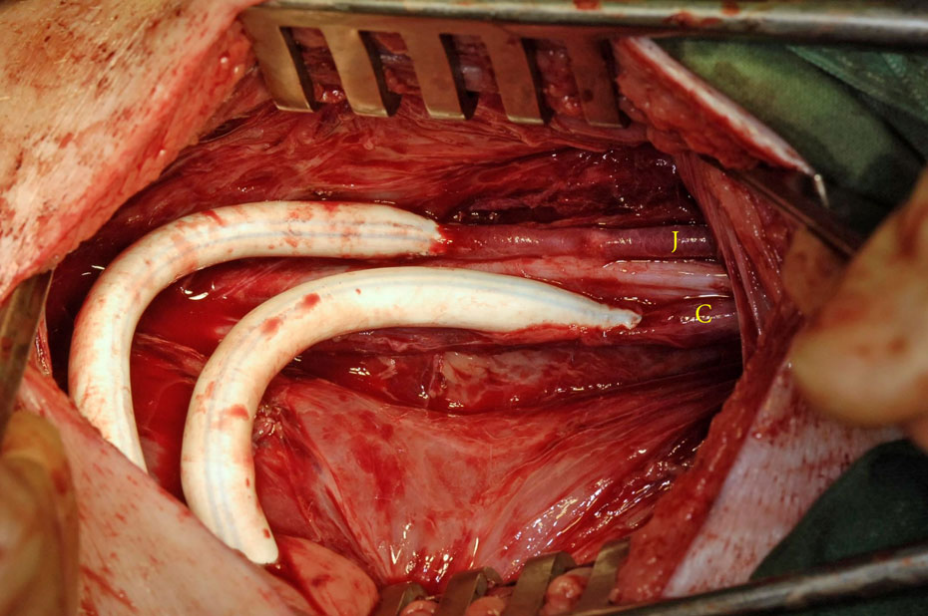
Crossover carotid jugular fistula showing a paired bilateral long length ePTFE AVG from the common carotid artery connected to the *contralateral*internal jugular vein.

**Figure 4:**
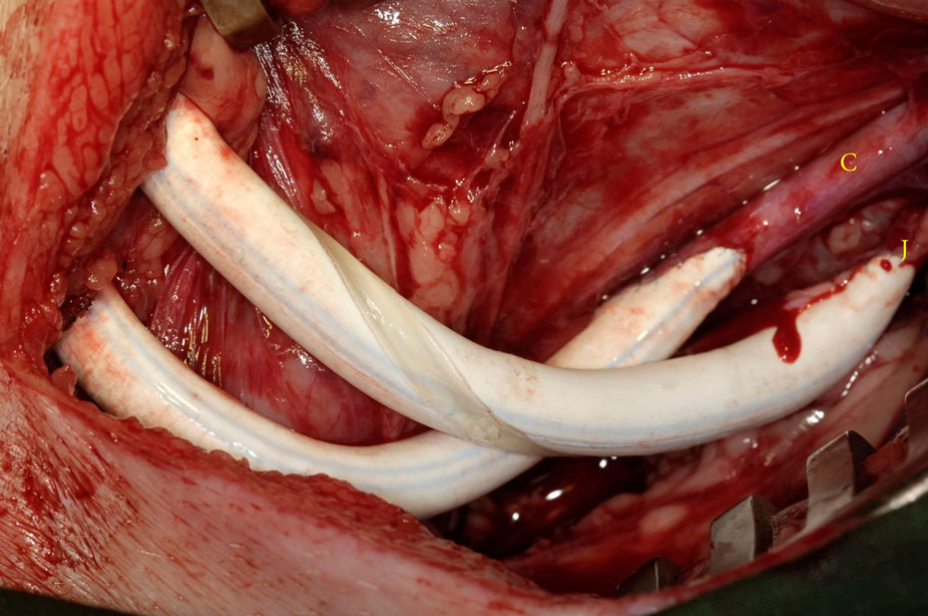
**The ePTFE control graft anastomosed to the common carotid artery**. The Spiral graft anastomosed to the internal jugular vein. J = jugular vein; C = carotid artery.

Through a longitudinal incision in the midline of the neck, the common carotid artery and the internal jugular vein were dissected bilaterally. Papaverine solution five mg (diluted with salin solution 1:10) was applied locally to prevent vascular spasm. All AVG were created by experienced vascular surgeons (A.Kh. J., G.J.d B). Coin tossing was done to determine which graft type was positioned on which arterial side. The graft length was standardized on 150 mm. Baseline diameter of the carotid artery was 4-5 millimeter, and approximately 5 millimeter for the jugular vein. In this animal model the sizing of both vein and artery are similar on the left and right side. The artery was occluded by vesselloops, and a 20-mm arteriotomy was performed. An end-to-side anastomosis was created at a 45° angle using a continuous suture of 7-0 prolene The venous anastomosis was created in a similar fashion (Figure 2, 3 and 4). Mean operating time including neck dissection and 4 anastomoses was 107 minutes ± 12 minutes. The animals were euthanized after 2 days (pig 1, pig 2), or 14 days (pig 3, pig 4) following control angiography through a femoral access.

## Results

**Figure 5:**
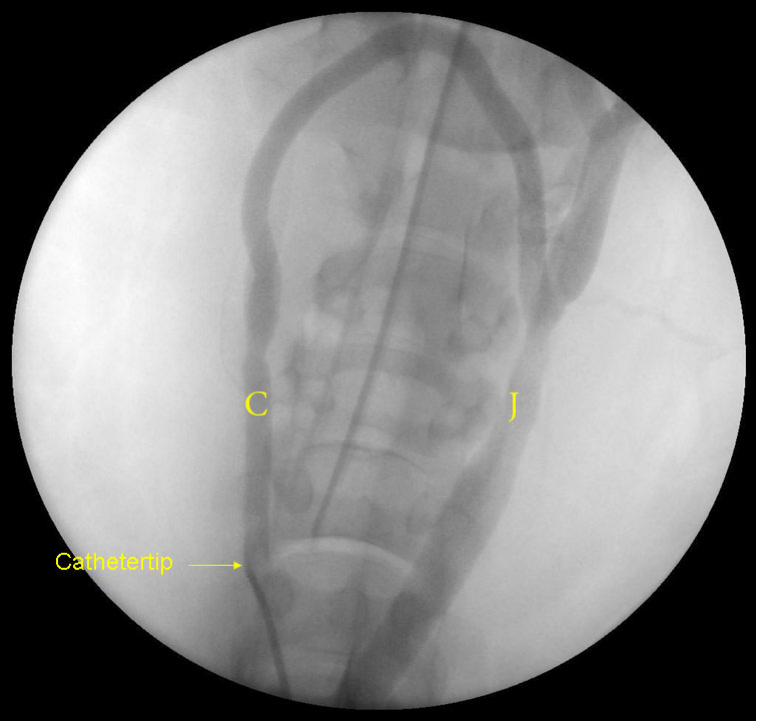
**Patent ePTFE crossover AVG**. We found no angiographic or macroscopic sign of narrowing at the anastomosis site. Arterial anastomosis at the right common carotid artery and the venous anastomosis at the left internal jugular vein. J = jugular vein; C = carotid artery.

At two days, both pig 1 and 2 showed patent carotid access grafts (both Spiral and Control grafts) (Figure 5). Pig 1 had normal patency of the jugular vein while pig 2 had limited (approximately 20%) luminal narrowing/spasm in the distal jugular vein, distal from the venous anastomosis based on angiography. At two weeks, both pig 3 and 4 showed patent carotid access grafts (both Spiral and Control grafts).

After exposure of the surgical site, dissection of the grafts showed satisfactory early surgical changes, no signs of infection, and intact anastomoses in all 4 pigs. Especially there was no kinking or impression of the AVG in the midline. In animal 3 and 4, after a 14 day follow-up, all grafts and adjacent vessels were excised and examined by longitudinal section before immersion in formalin for at least 24 h.

## Discussion

**Figure 6:**
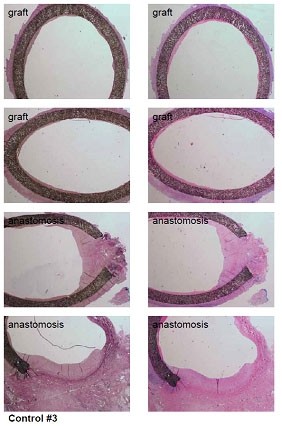
histologically prove of the model with IMH in both graft types after 4 weeks survival.

**Figure 7:**
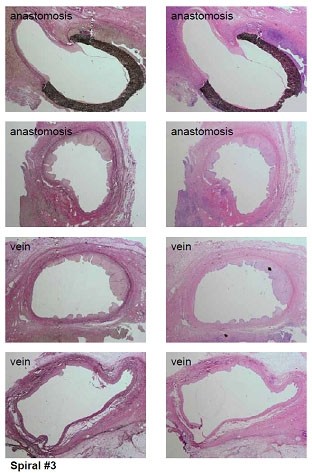
histologically prove of the model with IMH in both graft types after 4 weeks survival.

The advantages of this modified model include better simulation of human access graft model - i.e. graft length, loop fashion, orientation of anastomoses. An additional feature is the value of this model for access graft flow studies as the graft model allows better simulation of establishing flow effect of the longer loop model; better access for intraoperative Duplex ultrasound both proximally, mid, and distally in graft and jugular vein; the creation of a unidirectional and bidirectional outflow model as a means of better simulating the anatomical orientation of the preclinical model; all making this model more relevant to compare with the human access graft. This model results in IH (Figure 6 and 7). Although the formation of IH in this pilot study was not quantified, examples of histology where IH developed within the 4 weeks follow-up period can be obtained from our previous work [1,3].

For testing of intervention t the anastomsis site the same approach can be used as recently published by Huijbregts et al. [3]. To introduce a PTA balloon, the graft can be punctured using a standard needle with a Seldinger approach. Subsequently, a 5 or 6 French sheath can be introduced, and being used as the entrance for wires and balloons. Following intervention, the wires and sheath can be removed, and the puncture hole closed by using Prolene 6-0 suture.

Limitations. Even though the number of animals is small, the number seems to be sufficient for the purpose of the study, which is to assess the feasibility of a new surgical approach.

Several approaches can be chosen to study IH in animal models [4,5]. However, pigs are favorable animals to study cardiovascular disease because of their analogous vascular anatomy, size, and physiology. Furthermore, the rapid intimal hyperplasia formation at the venous anastomosis in our model closely resembles human hemodialysis graft failure [1,3]. On the other hand, 8 weeks after surgery a 50% patency loss can be expected in this model [1]. The maximum follow-up for this model therefore is recommended to be 4 weeks.

In the near future, this specific model will be used to further elaborate on the spiral flow effect possibly resulting in lower degree of IH formation.

This modified graft model for rapid AVG failure due to neo-intimal response at the venous outflow tract, offers an extended tool to study new graft materials, or to assess new strategies for improved AVG patency using local interventions at the venous anastomosis site.

## Conclusion

In this modified pig AVG failure model, implantation of a bilateral cross-over long AV graft between the carotid artery and contralateral jugular vein is a novel and feasible approach. The major advantage as compared to our previous model is that the present configuration allows the study of longer grafts and grafts with various design modifications, such as tapered or reinforced grafts. The present modified model offers a suitable tool to study local interventions or compare various graft designs aimed at improvement of AV graft patency

## Competing interests

The authors declare that they have no competing interests.

## Authors' contributions

AKhJ carried out the animal data, collected essential data, and drafted he manuscript. IH carried out the animal study, helped to collect and interpret data, and helped to draft the manuscript. FM participated in study design, helped to interpret the data, and approved the final manuscript. JH participated in study design, and helped to carry out he animal study. Performed language corrections for revised manuscript. PS participated in study design, and approved the final manuscript. PB participated in study design, helped to interpret data and to draft the manuscript. GB performed study design, carried out the animal study, drafted the manuscript, and carries overall responsibility for the contents of this manuscript. All authors read and approved the final and revised manuscript.
